# Intratesticular Leiomyoma: Lessons Learned From a Case Report and Another Contribution to Enlarge a Small World Series

**DOI:** 10.7759/cureus.35173

**Published:** 2023-02-19

**Authors:** Mohamed Amine Haouane, Fouad Hajji, Omar Ghoundale, Mohamed Amine Azami

**Affiliations:** 1 Department of Pathology, Faculty of Medicine and Pharmacy, Cadi Ayyad University/Ibn Sina Military Hospital, Marrakech, MAR; 2 Department of Urology, Cadi Ayyad University/Ibn Sina Military Hospital, Marrakech, MAR; 3 Department of Pathology, Cadi Ayyad University/Ibn Sina Military Hospital, Marrakech, MAR

**Keywords:** care guidelines, case report, frozen section, tumor, benign, testicle, leiomyoma

## Abstract

Leiomyomas are benign, slow-growing, mesenchymal neoplasms that originate from smooth muscle. We report a case of a 44-year-old man presented with an asymptomatic left scrotal mass. Scrotal ultrasonography (US) showed a 3.9 cm well-limited, hypoechoic intratesticular mass. The patient underwent a radical left orchiectomy and histologic findings revealed an intratesticular leiomyoma. To our best knowledge, so far, this is the 19^th^ case of intratesticular leiomyoma to be reported in the literature. Furthermore, intraoperative frozen section examination should have been part of the management armamentarium in this case, leading to enhanced testicular preservation.

## Introduction

Neoplasms of the testis are most often primary testicular tumors of germ cell origin, occurring most frequently between the ages of 25 and 35 years and are almost always malignant. Less commonly, non-testicular malignancies may involve the testis, including leukemia, lymphoma, and metastatic disease [1]. In the setting of suspected testicular mass, radical inguinal orchiectomy is almost always performed. It establishes the histological diagnosis and primary T stage, provides valuable prognostic information, and is curative in 80% to 85% of germ cell tumors (GCT) [2]. Nevertheless, benign lesions do also exist, and may include teratoma, benign sex cord-stromal tumor (Leydig and Sertoli cell tumors) and lipoma. They may be encountered in up to 80% of testicular lesions smaller than 3 cm with a long duration of symptoms (>6 months) [3], raising both diagnostic and therapeutic challenges [4, 5]. Herein, we describe a rare case of benign testicular tumor, which occurred in a 44-year-old man and proved to be an intratesticular leiomyoma on histopathology.

## Case presentation

A 44-year-old white man was referred by his general practitioner because of a clinical finding of a painless left scrotal mass. The patient had noticed a progressive, painless swelling in the left hemiscrotum over the past six months. He had two children, and denied any history of undescended testis, development sex disorders (DSD), trauma, weight loss or appetite disturbance. No family history of testicular malignancies was also noticed.

**Figure 1:**
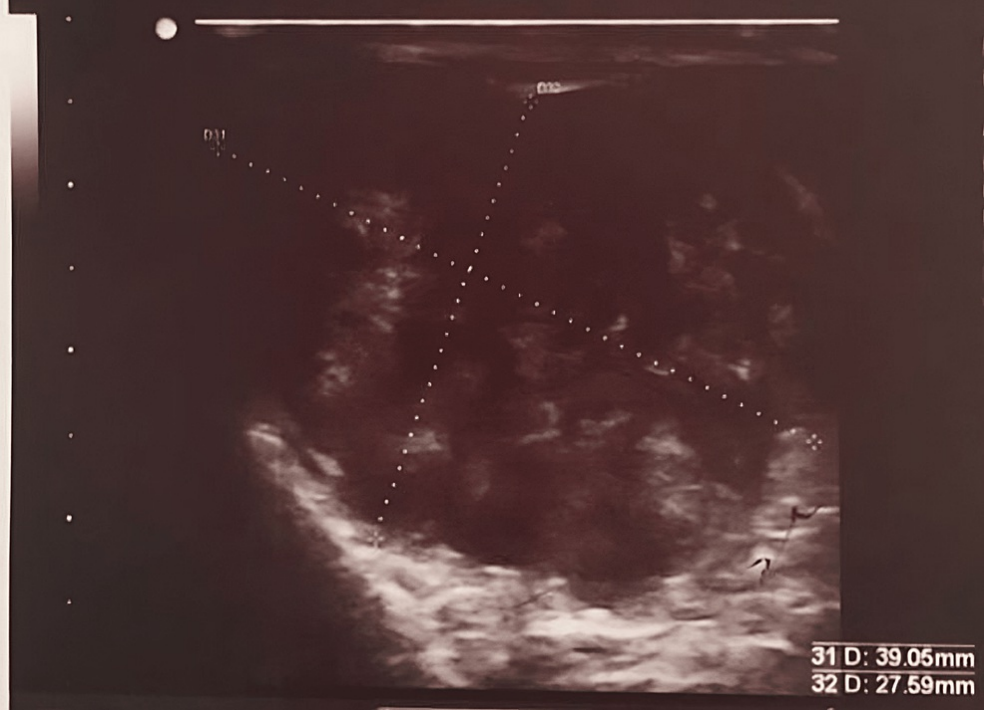
Scrotal ultrasonography (US) showed a well-limited heterogeneous hypoechoic intratesticular mass measuring 3.9 x 2.7 cm on the left testis.

On physical examination, he had a firm and painless mass measuring 4 cm × 3 cm covering the whole left testis, while his right testis was unremarkable. Serum tumors markers (alpha-fetoprotein and beta-human chorionic gonadotropin) showed no elevated levels. Scrotal ultrasonography (US) showed a well-limited heterogeneous hypoechoic intratesticular mass measuring 3.9 x 2.7 cm on the left testis, the rest of the testicular parenchyma appeared homogeneous and no hydrocele was mentioned (Figure 1).

The patient consented to undergo a radical inguinal left orchiectomy, whereupon the left testis was found to be enlarged with a palpable and firm intra-parenchymal mass. The patient’s postoperative recovery was uneventful.

**Figure 2:**
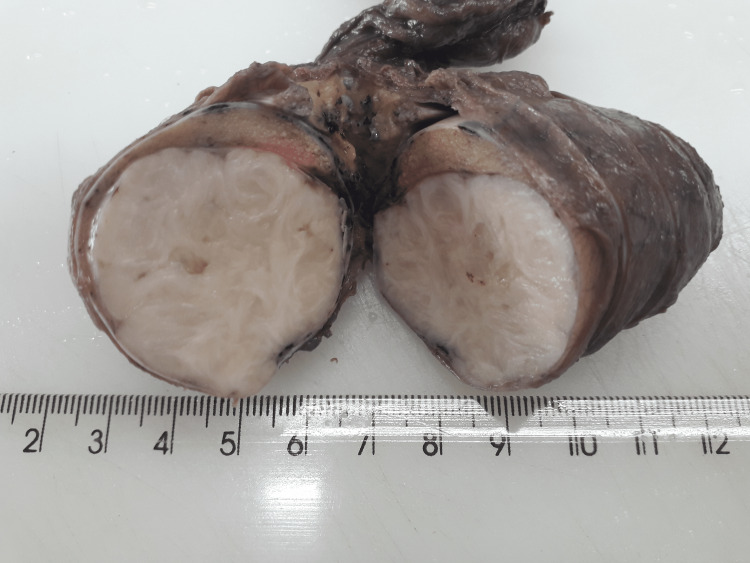
The gross examination of the specimen showed a whitish nodular mass that measured 4.2 cm in diameter, with a fasciculated aspect on section and well demarcation from adjoining uninvolved testis.

Pathologically, the gross specimen showed a whitish nodular mass that measured 4.2 cm in diameter, with a fasciculated aspect on section and well demarcation from the remainder testicular parenchyma (Figure 2).

**Figure 3:**
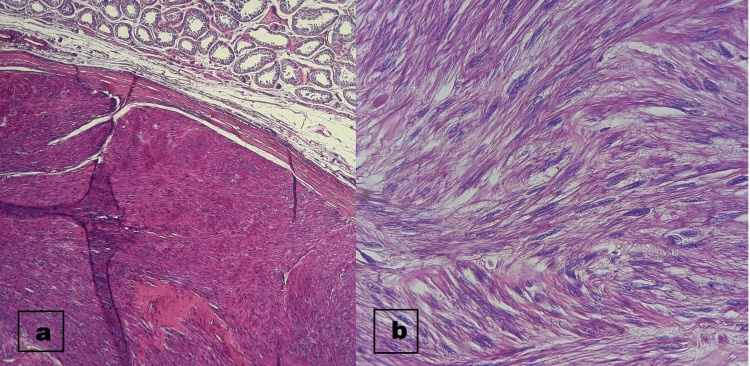
Histological section showed: a) Normal testicular tissue adjacent to a well-limited encapsulated mass (hematoxylin-eosin stain; original magnification x100). b) The tumor proliferation is formed by interlacing bundles of spindle elongated cells, with fusiform nuclei, rounded ends and eosinophilic cytoplasm with poorly defined cytoplasm limits (hematoxylin-eosin stain; original magnification x200).

Under the microscope, the tumor was composed of interlacing bundles of spindle cells, the cells were elongated, with fusiform nuclei, rounded ends and an eosinophilic cytoplasm with poorly defined cytoplasmic limits. Cytonuclear atypia was exceptional, mitosis and coagulative necrosis was absent (Figure 3).

**Figure 4:**
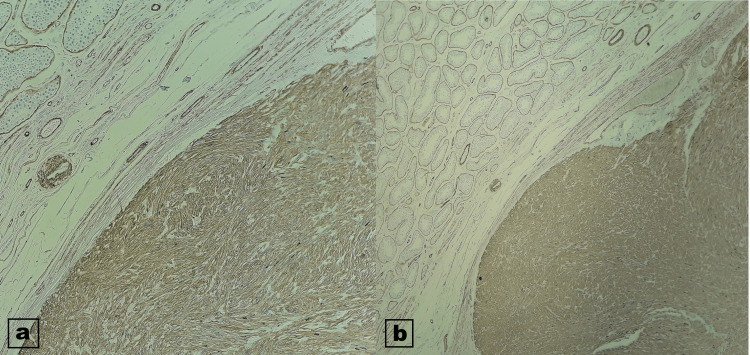
Immunohistochemical studies showed that the spindle cells were diffusely positive for smooth muscle actin (a) and desmin (b).

Immunohistochemical studies showed that the spindle cells were diffusely positive for smooth muscle actin (SMA) and desmin (Figure 4).

These histopathological findings were consistent with the diagnosis of leiomyoma without any sign of malignancy. At his four-year follow-up, the patient showed no signs of local recurrence nor distant metastasis.

## Discussion

Leiomyoma of the testis is an uncommon condition [6]. To our best knowledge, only about 18 cases have been reported to date in the English literature since the first case was described in 1972 [4]. Leiomyomas are soft-tissue benign tumors that develop from smooth muscle [6]. How this entity can come about remains controversial; however, it has recently been hypothesized that it arises from contractile cells in the seminiferous tubules [7]. Clinically, testicular leiomyoma is a non-tender solid scrotal tumor that expands in size. It is most common in males in their fourth and fifth decades of life [5,8]. It is sometimes associated with hydrocele [9]. The tumor biomarkers are, for the most part, normal [9]. Ultrasonography has long been the gold standard for evaluating testicular masses [5], and leiomyoma presents as a well-hypoechoic homogenous or heterogeneous mass with variable calcifications [10]. However, because it has the same radiological semiology as malignant tumors, which constitute the majority of testicular tumors, it cannot be diagnosed using ultrasonography or magnetic resonance imaging. As a result, the definitive diagnosis is established once the specimen has been examined histopathologically [10], and the treatment of choice is generally a radical orchiectomy [4-6,9,10].

However, a benign disease should have been considered in our patient who had presented with a long duration of symptoms and did not have any history of well-established risk factors for testis cancer (i.e., cryptorchidism, family or personal history of testis cancer, germ cell neoplasia in situ (GCNIS), and infertility), especially in the absence of disseminated GCT or elevated serum tumor markers. Indeed, the existence of a testicular mass should not be considered a sign of malignancy, and frozen section evaluation should be part of the treatment arsenal, which may lead to enhanced testicular preservation in particular cases [11]. The fact that leiomyomas are difficult to distinguish from malignancy on imaging and that the majority of testicular lesions are malignant justifies this approach [4,6]. Had the current case undergone intraoperative frozen section examination (FSE) of the intratesticular mass, testicular preservation approach would have been attempted as long as the specificity and sensitivity of intraoperative frozen section evaluation for the differential diagnosis of benign and malignant testicular lesions were excellent [12]. On gross pathologic examination, these tumors are well limited, firm, and have a whorled cut surface, with a variable presence of calcifications [6]. In terms of microscopic characteristics, intratesticular leiomyoma is characterized by cellular proliferation of bland spindle cells with cigar-shaped nuclei and eosinophilic cytoplasm. The diagnosis is confirmed by the immunostaining positivity for smooth muscle-specific actin and desmin. The prognosis is favorable, and there has been no evidence of recurrence or malignant transformation to date [10].

## Conclusions

In the setting of an indeterminate intratesticular mass, a benign lesion should be considered. However, leiomyoma is a rare benign testicular neoplasm that is diagnosed based on histopathological confirmation almost on a radical orchiectomy specimen. By reporting this case, we aim to stress the importance and reliability of FSE in the correct diagnosis of an indeterminate testicular mass. Perhaps a surgical management strategy for testicular masses based on FSE diagnosis can be implemented with the goal of increasing organ preservation surgery.
